# Supplementary material for: Effectiveness of targeted antenatal family planning information provision on early postpartum family planning uptake in Kisumu County: Protocol for a simple randomized control trial (I DECIDE Study)
Source: PLoS One. 2022 Aug 15;17(8):e0264807. doi: 10.1371/journal.pone.0264807 (PMC9377582; doi:10.1371/journal.pone.0264807)
Supplement: S1 Protocol — (DOC) [file pone.0264807.s003.doc]

| Section/item (Item No) | Description |
| --- | --- |
| **Administrative information** | |
| Title (1) | Effectiveness of targeted antenatal family planning information provision on early postpartum family planning uptake in Kisumu County |
| Trial registration (2a/b) | Pan African Clinical Trials Registry PACTR202109586388973 |
|  | **Primary registry and trial identifying number** Pan African Clinical Trial Registry  PACTR202109586388973  **Date of registration in primary registry** 28 September, 2021  **Secondary identifying numbers/Acronym** I-decide  **Source(s) of monetary or material support:** The PI (MSS)  **Primary sponsor**: The PI  **Secondary sponsor(s)**: None  **Contact for public queries**: MSS, BScN, MScN [+254720640142] [senghormorris@gmail.com]  **Contact for scientific queries**: MSS, BScN, MScN [+254720640142] [senghormorris@gmail.com]  **Public title**: Effectiveness of targeted antenatal family planning information provision on early postpartum family planning uptake in Kisumu county  **Scientific title:** Targeted Antenatal Information Provision and Postpartum Fertility Decisions  **Countries of recruitment**: Kenya  **Health condition(s) or problem(s) studied**: Female Fertility, Contraceptives  **Intervention(s)**  **Active comparator:** Antenatal provision of Postpartum Family Planning (PPFP) Information and appointment setting for 14 weeks postpartum follow-up  **Control comparator:** Routine Antenatal care  **Key inclusion and exclusion criteria** The study will be conducted in 4 primary health centres and 2 communities in the Kisumu East sub-county. The Health centres are eligible if : They offer the continuum of ANC, delivery, and PNC; They provide a selection of at least three modern contraceptive methods that are rated 2 or 1 on the Medical Eligibility Criteria (MEC) for Postpartum Contraceptives and referrals for other methods to clients; There were no stock-outs of contraceptives during the preceding six months; They have on average at least 10 deliveries per month; and They are willing to participate.  All pregnant women will be eligible to participate in the study if: They are in their second pregnancy trimester; The woman is attending ANC and has intention of attending PNC at the health centre; An informed consent is obtained; and Resides within 20km  Excluded from the study will be clients: in similar study; with latex sensitivity; not anticipating a male partner in the next 12 months; unable to complete consent form as determined by the study nurse or cha and whose only male partner has had a vasectomy.  **Study type** Interventional  **Allocation:** randomized  **Intervention model:** concurrent assignment  **Masking:** None  **Primary purpose:** prevention  Phase II  **Date of first enrolment** Nov 2021  **Target sample size** 246  **Recruitment status** Not Recruiting  **Primary outcome(s)** Nature of Fertility decisions postpartum. Primarily the uptake of immediate postpartum FP being the main outcome – 14 weeks postpartum  **Key secondary outcomes** Fertility intentions. Primarily whether they accept to book an appointment for postpartum family planning - 14 weeks postpartum  Knowledge of family planning methods – Any time after intervention |
| Protocol version (3) | 28^th^ September 2021 |
| Funding (4) | The Trial will be funded by the principal investigator. |
| Roles and responsibilities (5a/b/c/d) | Names, affiliations, and roles of protocol contributors  Morris Senghor Shisanya (PI)  School of Nursing, Kibabii University, Postal Address 1699-50200 Bungoma, Kenya  Email: [mshisanya@kibu.ac.ke](mailto:mshisanya@kibu.ac.ke)  Gregory Ganda (Co-PI)  Department of Health and Sanitation, County Government of Kisumu, Postal Address 721-40100, Kisumu Kenya  Email: [gregganda2@gmail.com](mailto:gregganda2@gmail.com)  Collins Ouma (Co-PI)  Department of Biomedical Science and Technology, Maseno University, Private Bag, Maseno, Kenya  Email: [couma@maseno.ac.ke](mailto:couma@maseno.ac.ke)  Kipmerewo Mary (Co-PI)  School of Nursing, Midwifery and Paramedical Sciences (SONMAPS), Masinde Muliro University of Science and Technology, Postal Address 190-50100, Kakamega, Kenya  Email: [kipmerewosoi@yahoo.com](mailto:kipmerewosoi@yahoo.com) |
|  | Name and contact information for the trial sponsor  Morris Senghor Shisanya (PhD Student)  School of Nursing, Kibabii University, Postal Address 1699-50200 Bungoma, Kenya  Email: [mshisanya@kibu.ac.ke](mailto:mshisanya@kibu.ac.ke) |
|  | The principal investigator developed the study design, data collection, management, analysis and interpretation for his PhD Thesis. He will be lead the process of report writing and publication. However, the study core team has the ultimate authority over conduct of the study. |
|  | The study core team will have a coordinating centre in Gita Sub county Hospital. The steering committee will be composed of the PI and the 3 CoPIs.  The steering committee has the oversight of Trial title, sourcing for funding, protocol amendment, follow-up on data collection and management recommendations by data management team, setting recruitment date, setting recruitment end date, monitoring actual recruitment rate versus the projected recruitment rate, consenting process, summarising protocol deviations, site visits and site reporting of organizational problems or other trial issues  Data management team will comprise the PI, Data Clerks at the sites and the study nurse and Community Health Worker: set acceptance rate proportion (those who accepted to participate versus those sampled), monitoring retention (percentage of participants proceeding from recruitment, treatment to follow-up including missing outcome data), monthly forecast of recruitment for the trial period remaining, monitoring loss to follow-up as a proportion of the ones not yet reached by 16 weeks postpartum follow-up to those who were recruited, overseeing data management metrics i.e. rate of electronic data capture, return dates and rate of returns, number of completed follow-up, withdrawal rate, monitoring intervention fidelity by the study nurse or Community Health Worker (CHW), Data quality checks, results dashboard review  The end point adjudication team will comprise the PI, the facility in-charges of the study facilities, the sub-county RH coordinator. |
| **Introduction** | |
| Background and rationale (6a/b) | **Problem Statement**  Postpartum women are at a high risk of unplanned pregnancies, especially in the first year after delivery (Moore et al., 2015). Studies have shown that the need for contraceptives varies during a woman’s reproductive years, but demand is highest during the postpartum period (Gebreselassie et al., 2008). Despite observed advances in access to RMNCH services in Kenya in recent decades, progress is feeble when it comes to effective postpartum contraceptive services integration and use (Chimbi, 2018). A demographic and health survey in Kenya (KDHS) found that only 25% of women in Kenya had adopted postpartum family planning (PPFP) by six months, and 35% at one year. The same KDHS, 2014 reveals that Nyanza region has the shortest median postpartum abstinence with one of the shortest inter-birth intervals nationally. Correspondingly, the mean number of children in Kisumu county by the time a woman is 25-40 years is 5.6. This is higher than the national average of 5(KNBS, 2015). Compared to other Counties, Kisumu County has low postpartum care provision including PPFP. The county has about 2/3 of the services being provided to other Counties that were studied and averaged about 80 (Tupange et al., 2011). These statistics are an indicator of either outcomes of postpartum care including PPFP or aspects that warrant need for acceleration of PPFP services in Kisumu County.  It is unknown why there is a low uptake of family planning amongst postpartum women despite their multiple contacts with healthcare providers in health facilities. Antenatal period and ANC pose such an opportunity for information giving, counselling and behavioural contracting for PPFP. However, there is no protocol on structured FP information giving, counselling and implied behavioural contracting to ensure earliest uptake to enhance appropriate desired inter-birth interval.  The multiple factors pertaining inclusion PPFP in RMNCH services have not been adequately studied. These factors can be examined from the perspective of the client’s (demand) and health care (supply) sides. The demand side factors include sociocultural barriers, individual perceived behavioural control and future intention of utilization. The supply side factors are; poor skills, staffing levels, waiting time, essential supplies, timely referral, cost, staff knowledge, respectful safe care, mistrust and staff interpersonal skills (Kyei-Nimakoh et al., 2016).  **Justification**  Routine Antenatal services offer frequent points of contact for providers and pregnant women. These contacts are vital opportunities to address the contraceptive needs of postpartum women (Al-Ateeq & Al-Rusaiess, 2015). PPFP should therefore be an integral part of Reproductive Maternal Neonatal and Child Health (RMNCH) services especially ANC. ANC FP counselling activities, provision of PPFP information, education, and counselling materials may have a positive effect on postpartum contraceptive uptake (Achyut et al., 2016). However, there is no structured, deliberate information giving integrated in ANC services as programmatic challenges (e.g., provider competencies, time pressure, and competition with other priority interventions) and policy barriers remain. This said, the evidence is often weak or incomplete and there are still knowledge gaps, particularly regarding studies that explore the desires, intentions, and priorities of women or couples related to PPFP, which may differ across settings and demography. Equally, there has been inadequate exploration of operationally-feasible ways to integrate PPFP information giving and counselling into existing ANC services. Further, there is a limited number of methodologically rigorous trials that give a detailed description of tested interventions and of how these were implemented.  The study will therefore examine the effectiveness of targeted antenatal family planning information provision on Postpartum Family Planning uptake by a rigorous randomised control trial. It will explore client (demand side) and health care system (supply side) of PPFP services as relates to ANC integration of information giving and fertility intention after delivery. KDHS 2014 fertility, FP and postpartum statistics for Kisumu County point to a PPFP provision gap. These aspects are; short postpartum abstinence period (≤2months), short interbirth intervals (<24 months), higher (5.6) than national (5) average number of children per woman between ages 25 and 40 years and low postpartum service provision including PPFP as compared to other counties and regions. Kisumu county, therefore, will be a suitable site for this study as it has adverse trends in postpartum care provision and outcomes. These two aspects can be adequately addressed by the study and will form a basis for conclusion as to whether the models of PPFP service provision proposed in the trial are clinically superior to the routine practice (County Government of Kisumu, 2019; KNBS, 2015; Tupange et al., 2011).  **Study Aim**  Broadly, the researcher intends to examine the effectiveness of targeted antenatal family planning information provision on early postpartum family planning uptake in Kisumu County. |
|  | Choice of comparators  The intervention is Antenatal provision of Postpartum Family Planning (PPFP) Information and appointment setting for 14 weeks postpartum follow-up. This intervention can be feasibly applied to the current Maternal and Child Health services in Kenya  The control comparator for this study is the routine care that is given to antenatal mothers. It was chosen because it is applied uniformly in the setting from which the participants were drawn. Secondly, it is ethically acceptable because the standard care will not be denied from the control group. Third is that it is a relevant comparator as it the actual services that the treatment is being compared to and bears close semblance to the intervention. Lastly, the comparator will control for threats to internal validity. |
| Objectives (7) | The researcher will specifically:   1. Evaluate effect of socio-cultural beliefs on Postpartum Family Planning uptake among postpartum mothers; 2. Assess perceived individual control of Postpartum Family Planning choice among postpartum; 3. Analyse fertility intentions for postpartum mothers after the intervention; 4. Compare Postpartum Family Planning uptake between control and intervention groups of postpartum 5. Examine the determinants of Postpartum Family Planning uptake between control and intervention groups of postpartum mothers in Kisumu County. |
| Trial design (8) | This will be a prospective interventional study, a cluster Randomised Control Trial (RCT) conducted in Kisumu County  The study will have three arms. These will be facility intervention arm, community intervention arm and a control arm. The study will have three interacting phases. The pre-intervention phase, intervention phase and post-intervention phase. The proposed methods in each phase are not complex thus the overall design can be classified as simple intervention design.  Pre-intervention phase is for establishing sampling frame, intervention package and research tools formulation.  The aim of the intervention phase is to determine the effect of the targeted antenatal PPFP information package on the uptake of contraceptive methods during the postpartum period, in comparison with the standard of care. The intervention phase also integrates qualitative research that is aimed at identifying operational barriers and enablers of the intervention outcomes.  Randomization to control and intervention arms is at individual level within randomly selected control, community and the primary level health centres. Participants allocated to the experimental group will receive the targeted antenatal PPFP information package and those allocated to the control group will receive usual antenatal care. |
| Methods: Participants, interventions, and outcomes | |
| Study setting (9) | The study will be in Kisumu County, Kisumu East sub County. The randomly sampled facilities are Migosi Health centre and Gita Health Centre for Intervention in urban and rural area respectively. The Community Units (CU) for community-based intervention will be Kuoyo CU and Nyalunya CU in in urban and rural area respectively. The control Facility and CUs will be Kowino HC and Chiga HC and their link CUs in urban and rural area respectively. |
| Eligibility criteria (10) | This study will be among pregnant mothers in their third trimester, irrespective of age, attending ANC clinic in the intervention facilities or are within the respective CUs followed up to 3 months postpartum. The study will be conducted in 4 primary health centres and 2 community units in Kisumu East sub county. The Health centres eligibility depended on if: they offer the continuum of ANC, delivery, and PNC; they provide a selection of at least three modern contraceptive methods that are rated 2 or 1 on the Medical Eligibility Criteria (MEC) for postpartum contraceptives and there were no stock-outs of contraceptives during the preceding six months |
| Interventions (11a/b/c/d) | The intervention will be; provision of antenatal information on PPFP using a standardized PPFP counselling tool and postpartum appointment setting. The control group will be under the routine standard antenatal care. There will be antenatal PPFP information provision training for the service providers at the facility and Community Health Assistants (CHA) for the community arm to standardize the intervention. The intervention will be administered in the second and third trimester of pregnancy. The study nurse or CHA will use a standardised tool of Medical Eligibility Criteria |
|  | Client can be discontinued if they lose their only sexual partner, the only sexual partner undergoes vasectomy in the course of pregnancy, develop postpartum psychosis, are hospitalized for more than 14 weeks postpartum. |
|  | Client exit interviews returns to data base and the dashboard will be reviewed by the data management team to give feedback to the study steering committee for appropriate corrective actions to improve adherence to intervention protocols |
|  | The is no concomitant care or intervention that is recommended or prohibited during the trial |
| Outcomes (12) | Primary Outcome  Nature of Fertility decisions postpartum. Primarily the uptake of immediate postpartum FP being the main outcome. To be measured at or after 14 weeks postpartum. It is a proportion.  Secondary Outcome  1. Fertility intentions. Primarily whether they accept to book an appointment for postpartum family planning. Will be assessed soon after the intervention has been administered. It will be a proportion of those intending to use PPFP versus those who participated.  2. Knowledge of family planning methods. Will be assessed soon after the intervention has been administered or recruitment (for controls). It will be a proportion of level of knowledge on PPFP among participated.  3. Uptake of postnatal services. Will be measured at 14 weeks postpartum and it will be a proportion of utilization of postnatal services among participants.  4. Perceived control of fertility decision. Will be a proportion among participants. To be measured soon after intervention or recruitment (for the controls)  5. Attitude toward PPFP Will be a proportion among participants. To be measured soon after intervention or recruitment (for the controls)  No harm outcomes are premeditated |
| Participant timeline (13) | This being a behavioural intervention, the recruitment and interventions will be done simultaneously.  Client exit interviews will be done immediately or soonest possible time after the intervention  Questionnaire will be administered at or soon after 14 weeks postpartum.  (see annexes 1 and 2)  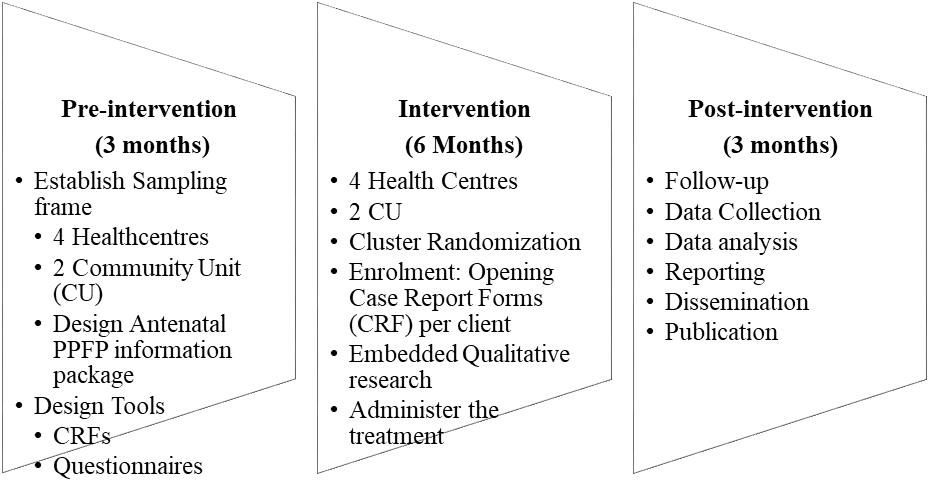 |
| Sample size (14) | The sample size is estimated based on the following assumptions: among women at three months postpartum, KDHS data report 27% of use of any method (modern or traditional) in Kenya while the CPR in the general population is 53% (KNBS, 2015) . These figures allow the assumption of a desired 26% difference between control and the intervention groups (26% increase in adopting a modern contraceptive by three months postpartum). Therefore the sample size was calculated pairwise for two separate RCT for community arm and the control and the facility arm and the control (Fleiss et al., 2004; Freidlin et al., 2008; Kane, 2018; Rosner, 2015). Rosner, 2015 proposed the sample size determination formula for difference in proportions with consideration of type I and II errors and power (Rosner, 2015; Suresh & Chandrashekara, 2012), N1={z_(1-α/2)*√(p ̅*q ̅*(1+1/k) )+z_1-β*√(p1*q1+(p2*q2)/k)}^2/Δ^2  Where q1=1-p1, q2=1-p2, p ̅=(p1+kp2)/(1+K) , p1, p2 = proportion (incidence) of groups #1 (27%) and #2 (53%) , Δ = |p2-p1| = absolute difference between two proportions (0.26), n1 = sample size for group #1, n2 = sample size for group #2, α = probability of type I error (is set at 0.05), β = probability of type II error (is set at 0.1 i.e. 90% power), z = critical Z value for a given α or β(1.96) and K = ratio of sample size for group #2 to group #1(1). Thus for practical equal sample distribution, the actual sample size shall be 246. Each facility shall have 41 clients |
| Recruitment (15) | Each health centre and community unit will have a trained research assistant. The assistant is to ensure adherence to the study manual and standard operating procedures for data management. The primary outcomes will largely be assessed based on the CRFs, appointment card and questionnaire. Actual uptake of modern contraceptive method will be established three months postpartum between the 12^th^ to 14^th^ weeks after birth. The CRFs will be filled on recruitment by the trained ANC service provider. The appointment card will be filled by the health worker after the client has accepted to set postnatal follow up date for PPFP. The questionnaire will be filled at 14 weeks postpartum during the scheduled MCH visit by a trained enumerator.  The client exit interview guide and site visit appraisal form will be used to assess the process indicators. Client exit interviews will be done by trained enumerator on the earliest opportunity after the intervention has been administered to reduce recall bias by the client. Site appraisal form will be filled on site visits by the research team and on the part of enumerator will be filled each day to include workload for ANC, number of staff available to offer ANC services, availability of FP counselling bag and flipchart. Lose to follow-up will be called to find the status of the client |
| **Methods: Assignment of interventions (for controlled trials)** | |
| Allocation: |  |
| Sequence generation (16a) | Each client meeting the criteria will be randomly assigned to the study i.e. to the intervention and to the control arms using simple random sampling by picking folded paper labelled “yes” or “no”. There will be no blinding as the intervention facilities and controls are known and will be separate to reduce contamination. |
| Allocation concealment mechanism (16b) | There will be no allocation concealment as the intervention and control facilities are known and separate to reduce contamination. |
| Implementation (16c) | The allocation to intervention and control is at the facility level. The participants will be enrolled by the study nurse for both the community and facility arms. Participants will be assigned to participate by picking a ballot paper labelled “yes” or “no” enclosed in resembling envelopes |
| Blinding (masking) (17a,b) | None of the implementers and participants will be blinded thud there equally no plan for controlled unblinding. |
| **Methods: Data collection, management, and analysis** | |
| Data collection methods (18a,b) | Each health centre and community unit will have a trained research assistant. The assistant is to ensure adherence to the study manual and standard operating procedures for data management. The primary outcomes are; PPFP knowledge, the intent for use of PPFP (behavioural contracting) and the actual uptake of modern contraceptive methods at three months postpartum will largely be assessed based on the CRFs, appointment card and questionnaire. Actual uptake of modern contraceptive method will be established three months postpartum between the 12^th^ to 14^th^ weeks after birth. The CRFs will be filled on recruitment by the trained ANC service provider. The appointment card will be filled by the health worker after the client has accepted to set postnatal follow up date for PPFP. The questionnaire will be filled at 14 weeks postpartum during the scheduled MCH visit by a trained enumerator.  A process evaluation will be undertaken with the objectives of understanding the barriers and enablers related to the delivery of PPFP. This will be evaluated based on client exit interviews and site appraisal forms. The client exit interview guide and site visit appraisal form will be used to assess the process quality indicators which will include: waiting time, time/trimester of start of FP counselling, time after FP counselling, group/ individual session, availability of teaching aid on the table during counselling, gauging FP information level, application of GATHER (Greet, Ask, Tell, Help, Explain and Return/Refer) Model and BRAIDED (Benefits, Risks, Alternatives, Inquiries, Decision, Explanation, Documentation) Model. Satisfaction with information given, responses to questions, respectful care, confidentiality and privacy. Client exit interviews will be done by trained enumerator immediately after the intervention has been administered to reduce recall bias by the client. Site appraisal form will be filled on site visits by the research team and on the part of enumerator will be filled each day to include workload for ANC, number of staff available to offer ANC services, availability of FP counselling bag and flipchart. |
|  | Participant retention and complete follow-up will be promoted by ensuring that, first, the intervention and follow up are not so far apart and the there is no none routine appointment i.e. the follow-up appointment is set on the second immunization and postpartum care visit. Secondly, part of the inclusion criteria is distance of not more than 20 Km thus increasing access. Third is that the data collection forms have been converted to Kobo tool box with strict skip logics and restrains for required data. This is to ensure completeness of the data.  Diary of the return date of 14 weeks postpartum will be kept with telephone number to call the client up to 2 days after the material date |
| Data management (19) | **Data collection Procedure**  Five tools will be used for data collection, namely; client exit interview guide, case report form, appointment card, Site appraisal form and questionnaire. All the tools will be used to collect quantitative data except site appraisal form and some questions in the questionnaire that need brief explanation. The theory of planned behaviour was applied to design quantitative process and outcome indicators and thus the tools (Ajzen & Klobas, 2013). Client exit interview guide and Site appraisal form will be developed based on the procedures set out in the counselling guide. Appointment card will be source of information on client details, proposed date for PPFP initiation and vital PPFP information summary.  **Data collection**  Each health centre and community unit will have a trained research assistant. The assistant is to ensure adherence to the study manual and standard operating procedures for data management. The primary outcomes are; PPFP knowledge, the intent for use of PPFP (behavioural contracting) and the actual uptake of modern contraceptive methods at three months postpartum will largely be assessed based on the CRFs, appointment card and questionnaire. Actual uptake of modern contraceptive method will be established three months postpartum between the 12^th^ to 14^th^ weeks after birth. The CRFs will be filled on recruitment by the trained ANC service provider. The appointment card will be filled by the health worker after the client has accepted to set postnatal follow up date for PPFP. The questionnaire will be filled at 14 weeks postpartum during the scheduled MCH visit by a trained enumerator.  A process evaluation will be undertaken with the objectives of understanding the barriers and enablers related to the delivery of PPFP. This will be evaluated based on client exit interviews and site appraisal forms. The client exit interview guide and site visit appraisal form will be used to assess the process quality indicators which will include: waiting time, time/trimester of start of FP counselling, time after FP counselling, group/ individual session, availability of teaching aid on the table during counselling, gauging FP information level, application of GATHER (Greet, Ask, Tell, Help, Explain and Return/Refer) Model and BRAIDED (Benefits, Risks, Alternatives, Inquiries, Decision, Explanation, Documentation) Model. Satisfaction with information given, responses to questions, respectful care, confidentiality and privacy. Client exit interviews will be done by trained enumerator immediately after the intervention has been administered to reduce recall bias by the client. Site appraisal form will be filled on site visits by the research team and on the part of enumerator will be filled each day to include workload for ANC, number of staff available to offer ANC services, availability of FP counselling bag and flipchart.  Internal consistency will be ensured by piloting the tools and refining them to ensure they capture the essence of what they were meant to collect and Cronbach’s alpha of 0.7 will be acceptable. |
| Statistical methods (20a,b,c) | Quantitative data entry will be done in IBM’s SPSS version 26. The sub-Country research teams will be responsible for verifying the data and a second verification will be done by the researcher to monitor data quality. Questions about data inconsistencies or missing values will be sent to sites and will be resolved on an ongoing basis. The participant will form the unit of analysis and intra-cluster correlation coefficient will be accounted for. All analysis will be by intention to treat Descriptive statistics will be performed by computing means, standard deviations, and minimum and maximum values for continuous variables, and frequencies and percentages for categorical variables. As part of quality control and descriptive analysis of the data, the distribution of variables to detect outliers will be examined. Descriptive statistics will be tabulated for individual clusters and aggregated across clusters.  On evaluating the effect of socio-cultural beliefs on Postpartum Family Planning uptake among postpartum mothers in Kisumu County, descriptive statistics on of prevalent sociocultural beliefs about PPFP will be summarized into frequencies and percentages. Bivariate analysis of effects of sociocultural beliefs on PPFP uptake will be done and presented on two-by-two (2x2) tables with Chi-square being the inferential statistics where P-value will determine the significance of homogeneity of proportions and odds ratio (OR) and 95% confidence interval (95% CI) will demonstrate the strength of the relationship. Binary logistic regression analysis will be done to adjust for confounders of sociocultural beliefs as determinants of PPFP uptake  Perceived individual control of Postpartum Family Planning choice among postpartum mothers in Kisumu County will be analysed using descriptive statistics aggregated based on sociodemographic aspects and presented in tables with means, median, range and standard deviation. This will further be analysed by student t-test to ascertain the significance in differences in means for the determinants of perceived control of family planning choice Significant determinants will be fitted in multilinear regression analysis to adjust for confounders  Fertility intentions for postpartum mothers will be disaggregated based on sociodemographic aspects and other individual characteristics. Intention to use PPFP will be simplified in proportions of the categorical yes or no to appointment for PPFP. Bivariate analysis of determinants of fertility intentions will be done and presented in two-by-two (2x2) tables with Chi-square being the inferential statistics. Binary logistic regression analysis will be done to adjust for confounders of determinants of fertility intentions.  Likewise, level of intention will be measure by Likert scale and analysed by t-test statistics after normality testing and adjusted for in linear regression analysis.  Postpartum Family Planning uptake between control and intervention groups of will be compared by use of simple clinical superiority in the proportions.  Bivariate analysis with chi-square statistics will be used to analyse the determinants of Postpartum Family Planning uptake between control and intervention groups and thus will form the selection criteria for inclusion in final regression model. Binary logistic regression analysis will be applied to adjust for confounders |
|  | There are no additional methods proposed in this protocols. Any amendments will be communicated. |
|  | Intention to treat will be applied to protocol non-adherence.  Automatic linear model regression multiple imputation with 5 iterations with history for comparison of variance will be applied in handling of missing values. |
| **Methods: Monitoring** | |
| Data monitoring (21a,b) | Data management team will comprise the PI, Data Clerks at the sites and the study nurse and Community Health Worker: set acceptance rate proportion (those who accepted to participate versus those sampled), monitoring retention (percentage of participants proceeding from recruitment, treatment to follow-up including missing outcome data), monthly forecast of recruitment for the trial period remaining, monitoring loss to follow-up as a proportion of the ones not yet reached by 16 weeks postpartum follow-up to those who were recruited, overseeing data management metrics i.e. rate of electronic data capture, return dates and rate of returns, number of completed follow-up, withdrawal rate, monitoring intervention fidelity by the study nurse or Community Health Worker (CHW), Data quality checks, results dashboard review |
|  | The interim analysis will be for monitoring purposes from the Kobo toolbox dashboard. It will be used to determine acceptance rate and retention rate and forecasting trial period remaining and the proportion of the unreached or dropout rate. This being a behavioural intervention study, the researcher did not envisage a circumstance warranting prior termination of the trial due to internal factors. |
| Harms (22) | The study doesn’t envisage adverse events as it is a behavioural interventional study seeking to augment a vital preventive service. |
| Auditing (23) | The trial will be audited on monthly basis. The core and site trial team plus the facility in-charge and Sub-county RH coordinator will be involved in the auditing. |
| Ethics and dissemination | |
| Research ethics approval (24) | The study has undergone ethics review by Masinde Muliro University of Science and Technology (MMUST), (MMUST/IERC/013/2021) and has been licensed by the National Commission for Science, Technology and Innovation (NACOSTI), Ref. No. 522628. Informed consent will be sought before participant can be recruited |
| Protocol amendments (25) | Plans for communicating important protocol modifications (eg, changes to eligibility criteria, outcomes, analyses) to relevant parties (eg, investigators, REC/IRBs, trial participants, trial registries, journals, regulators)  Protocol amendments will be communicated by the PI to the Pan-African Clinical Trials Registry (PACTR), British Medical Journal (BMJ) (Journal for Trials), MMUST Institutional Ethics Review Committee (IERC) and NACOSTI |
| Consent or assent (26a,b) | The trials nurse at each site will obtain the consent from the trial participants |
|  | There will be no need for additional consent provisions to use individual participant’s data and biological specimens as the study will not publish individual participant data but anonymous group data and there are no biological specimen being collected. |
| Confidentiality (27) | Personal information about potential and enrolled participants will be collected using kobo tool box and submitted by the study nurse or CHW to the study server. Individual participants will be allocated randomly generated enrolment numbers to maintain anonymity all through the trial period. Access to data will be password encrypted. |
| Declaration of interests (28) | GG is the Chief officer of Health in Kisumu County but is not directly involved in care provision of the essential services to the study participant. The county government of Kisumu nurses employed in the facilities and the Community Health Assistants (CHA) will be the implementers of the intervention. All other core team members declare that they have no conflict of interest. |
| Access to data (29) | Access to trial dataset will be controlled. Request to be done by email to the principal investigator The decision is by the Research team lead by the Principal investigator. Criteria for reviewing the request: Qualification of the person requesting, description of the purpose of the request, willingness to engage the Principal investigator in the development of analysis plan, and monographs or manuscripts. |
| Ancillary and post-trial care (30) | No harm is anticipated in this trial thus no defined ancillary and post trail compensation. |
| Dissemination policy (31a,b,c) | The analysed results of this trial will be published in peer reviewed journal, Masinde Muliro University of Science and Technology (MMUST) thesis repository. There will be a feedback session with health care professionals, data will be shared upon reasonable request. |
|  | The core research team shall develop manuscripts for publishing. |
|  | The protocols with be published in peer reviewed trials journal, but participant level data sets and analysis code can be shared upon reasonable written request. |
| Appendices | |
| Informed consent materials (32) | Model consent form and other related documentation given to participants are available. |
| Biological specimens (33) | There will be no biological samples being collected for the purpose of this study |
