# Supplementary material for: Effectiveness of targeted antenatal family planning information provision on early postpartum family planning uptake in Kisumu County: Protocol for a simple randomized control trial (I DECIDE Study)
Source: PLoS One. 2022 Aug 15;17(8):e0264807. doi: 10.1371/journal.pone.0264807 (PMC9377582; doi:10.1371/journal.pone.0264807)
Supplement: Attachment — Submitted filename: Response to Reviewers 7.5.22.docx [file pone.0264807.s004.docx]

**Reviewer #1:** *The authors have come with an interesting research topic that touches women’s health, paving a way to addressing the unmet contraceptive needs in women living in resource-limited regions. In general, the study protocol is well written, and it should be considered for further research undertaking provided that the suggested modifications from both the reviewer (s) and academic editor(s) are incorporated adequately. ***Thank you for the time taken to read through the protocol. The manuscript has been updated to reflect reviewers’ suggestions.***

Below are the main comments of the review report.

*The researchers have declared that purpose of the study will be explained to the participants. I was wondering of a possibility of Hawthorne effect in which people behave differently (increased postpartum family planning uptake) once they know that they are being studied. **L279 Corrected to read introduced to the study**

*The objective of the study (study aim) was added to the methods section of the protocol. Is that appropriate? **L123-131 Placed before the methods section**

*How are the possibility of spillover effect and selective attrition will be addressed? **The possibility of spillover was catered for in allocating facilities to control and intervention arm thus limiting contamination. Secondly, at analysis level, intention to treat will be applied with multiple imputation estimation for endpoints.**

How is the influence of external factors such as mass media and partner’s desire are considered to be handled? **Theory of Planned Behaviour (TPB) (see S5L189) as adapted for the study has the background factors like mass media as source of information and normative beliefs as spousal/ in-laws perceptions about family planning being catered for. The questionnaire has captured these aspects.**

*There are some punctuation errors. For instance, the word ‘respectively’ is not preceded with a comma throughout the document. The word ‘antenatal’ was capitalized inappropriately. **L136-139 Corrected**

*Study aim section: the researcher or the researchers? **Corrected L124-131**

*List of abbreviations/acronyms should be revised. Abbreviations such as SGS, GATHER, BRAIDED should not be mentioned in the list as each one of them have been used only once and under brackets. **Corrected: Removed**

Reviewer #2:

Dear PLOSE ONE team of editorials, thanks for the chance given to me to review a protocol manuscript titled “Effectiveness of targeted antenatal family planning information provision on early postpartum family planning uptake in Kisumu county: Study protocol for a Simple Randomized Control Trial”. The protocol will have its own importance for future better utilization of the early post partal family planning and maternal health in general. The following are my comments for further improvement of the protocol.

1. General Comments

• The problem statement should be very strong to ensure the need to study ANC FP information provision to improve early PPFP uptake. The problem statement should be in line with purpose statement. **Corrected in L89-101.**

• The justifications of the study are not justifications – **Corrected L103-110**

• Try to shorten and summarize the analysis section. **Done**

• The phases and its intended objective should be clear and concise. **Done Fig 1 included L129**

• Are pregnant women best candidate for receiving FP information? How do you manage regret, depression or anxiety if they became pregnant by the failure of family planning? - **The introduction part has been improved to provide information on why the pregnant women are the right candidates…The ANC period as proposed by Achyut et al., (2016) L88-89 is appropriate for information provision to enhance early PPFP uptake. Method failure is one of the areas explored in the final questionnaire. Therefore, it is part of what will be measured.**

• It should be ‘clustered randomized trial’. **Considered, however, clustering of the facilities was on functional basis but actual randomization to participate or not to participate occurred among the clients visiting the facilities for antenatal services. Secondly, sample size calculation was for a simple RCT not for cluster RCT.**

2. Specific Comments

A. On The Methods Section

♣ What is unique in standardized intervention for at ANC FP information provision to improve early PPFP uptake when compared with routine one? **Standardization of the intervention is to be achieved through training of the information providers i.e. the nurse and the community health volunteer (CHV) and using the information provision tool L142 and L144-146. However, the standard care is the routine care offered in routine ANC. As highlighted in the problem statement the standard/ routine ANC doesn’t prioritize FP information provision.**

♣ Describe briefly the illegibility criteria i.e. If similar study is running in the health center? **Done L150-152**

♣ What type of family planning? I guess it should be reversible long acing contraceptive to have public health significance and to lengthen the birth interval? **The study does not exclude a method as long as it is described in the Medical Eligibility Criteria (MEC) of the WHO. The information provided is geared towards right usage and will be tailored to client preferences to avoid pitfall of predetermined choice by the provider.**

♣ How do you analyze fertility intentions? **The theory of planned behaviour (TPB) proposed by Ajzen is one of the widely tested theories and Azjen et al have proposed clear analysis of intentions which were adapted for the current research L250 added reference that provided the guideline. See page 11 section 4.2 Method 2: Generalized intention**

♣ Is the sample adequate to ensure external validity? **L174-184 the sample size calculated based on (Fleiss, Levin and Paik, 2004; Freidlin et al., 2008; Rosner, 2015; Kane, 2018). Being a simple RCT Sample size was calculated pairwise for each intervention arm and control with clinical superiority of a minimum desired difference of uptake (proportion) between the intervention and control groups being 26%, power 90%.**

♣ What are the exclusion criteria’s? **Included L150-152**

♣ What is the main purpose of the protocol development and what type of protocol is that? It is effectiveness. Hence, what are the pitfalls in detail for the low effectiveness of ANC FP provision to improve early uptake of PPFP?

♣ Who are those information conveyers? What is their special quality? **The information conveyor are nurses and CHVs working and linked in the participating facilities, respectively.**

♣ What are the contents of the counselling tool [try to mention by table]? **Done L143**

♣ What are the frequencies of conveying this advice? **Once**

♣ When do we say early post-partum? **Operationalized as the period from child birth to 3 months after birth. Typically, the period within which the mother returns to menses.**

♣ When do we say post-partal women have early PPFP uptake? Who measures that?  **Early PPFP uptake will be considered for those who will have started FP before lapse of 14 weeks postpartum. This will be measured by the researchers between 14-16 weeks postpartum using the final questionnaire.**

♣ What extent of early PPFP uptake do you expect as a result of your intervention?

Even did early PPFP uptake impact, outcome or an output? **The Uptake of early PPFP is the outcome. The expectation would be a clinically significant difference in early PPFP uptake between intervention and standard ANC.**

♣ How do you assess whether your project or other competing programs have impacted uptake of PPFP? **The exclusion criteria exclude eligible clients who are participating in other similar intervention like Immediate Postpartum Intrauterine Device (IPPIUD) etc. Similarly, randomization at client level is to avoid selection bias. Further, the intervention arms and control arm facilities are difference to reduce contamination and spillover effect.**

♣ Did the feasibility will follow the protocol? If so what expected results will derive you to continue to the next phase? **This being a behavioural intervention rather than a biomedical intervention seeks to determine the best model of Antenatal integration of the intervention (information giving to enhance FP uptake). Information giving is not a novel intervention rather it is known to work in improving FP uptake and is usually coupled to FP services provision. Therefore, the researchers’ piloting of the tools for operational feasibility is not likely to determine the sequelae of subsequent stage but to improve implementation and the tools used in the research.**

♣ Why don’t you use ecological framework? Have you consulted normalization theory? Is your comprehensive to cover all the stake holders of the post-partal family planning services actors? **We appreciate the suggestions and have considered them against the Theory of Planned Behaviour (TPB) which was adapted, conceptualized and operationalized for the current research. The suggestions have indeed enriched our thinking greatly, however, since the study is on effectiveness on uptake and the individual and social factors that may influence uptake, the TPB that has been adapted, and not simply adopted, best fits the interest of the researchers. Included in the TPB are background factors among them structural integrity (process fidelity) and others.**

♣ Simply what is your unit of analysis?  **The unit of analysis is the client and all statistical analysis will be based on the client.**

♣ What is your ratio of matching? **This being an RCT there will be no matching either during allocation or analysis because random assignment based on yes or no lottery system was used with the Non-Interference/Stable Unit Treatment Value Assumption thus assumed estimation of zero in sample variance. Therefore, if we were to assume a matching ratio then it would be 0.5 affording participants 50% chance of random allocation to either participating or not participating within each of the arms or treatment or control to each of the arms.**

♣ How did you assure data quality, mention in detail since it is plan? **Data quality will be assured through use of validated KOBO collect forms for recruitment, counselling/process guide, exit interviews and final questionnaire with proper skip logics. Secondly, the sub-Country research teams will be responsible for verifying the data and a second verification will be done by the researcher to monitor data quality. Questions about data inconsistencies or missing values will be sent to sites and will be resolved on an ongoing basis.**

♣ Why you prefer to use t-test? **T-test will be used for perceived individual control by the client and for fertility intentions. These two will be measured by Likert scale that will be converted to a continuous scale as describe in the TPB prototype analysis plan L246-249.**

♣ Generally, it is very good work with ample benefit that need brief refinement. **Thank you for your infallible inputs.**

Regards,

Morris Senghor
