# Supplementary material for: Effectiveness of targeted antenatal family planning information provision on early postpartum family planning uptake in Kisumu County: Protocol for a simple randomized control trial (I DECIDE Study)
Source: PLoS One. 2022 Aug 15;17(8):e0264807. doi: 10.1371/journal.pone.0264807 (PMC9377582; doi:10.1371/journal.pone.0264807)
Supplement: Attachment — Submitted filename: Response to Reviewer comments 5.6.22.docx [file pone.0264807.s005.docx]

**Thank you for the insights shared. Pleas find our responses below. Reviewer’s concerns are in bold and the responses are in normal font.**

The following are my comments;
• **Gaps related to the information need and the justification should be strong –** This has been noted, however, operational feasibility of integrating information giving during the antenatal period is the main intent of the study. Information as it were is already a proven means of increasing demand for FP but the main issue is how best should information be integrated in MCH services. Can deliberate antenatal integration be much better than routine way of doing it?

• **What are the adverse consequences occurred due to the convey of the conventional [routine standard care] post-partum family planning information? –** Study’s aim to compare the two Standard/routine care vs ANC Integrated PPFP information giving. The reason for comparison is because routine care has resulted/posted low uptake of early PPFP. L79-86

• **The eligibility criteria are not very strong and binding** – Noted. This is a behavioural intervention and basically applying implementation science as opposed to biomedical science. This means that apart from exclusion criteria, we used WHO FP eligibility criteria for inclusion.

• **The tool needs face validation -** Attached as S3

**• The protocol should address;**
i. **Clear sampling without making ambiguity to the reader** – Done L165-166, &L230-232

ii. **What are the software’s that are used to calculate the sample size and population allocation –** based reference 21 L492/493 A simple excel based calculator was used to calculate the sample size based on reference 19 L487/488.

iii. **Face validity of the tool** – See S2 attached

iv. **Pre-test** – Done

v**. The power and the effect expected due to the intervention/s** – Considered in formula as β (type II error set at 0.1 or 90% power) L181/182

vi. **Correction, revisions and actual effective implementations** – Were stipulated in the protocol registration with PACTR. So far no revisions have been made to the protocols registered with PACTR

vii. **Data collectors, their quality and data advanced data quality measures and its management** –Done L186-210, & L249-263

viii. **Dose, dosage, total duration of the intervention for both the case and the controls** – Done.L186-210

ix**. Expected outcomes**
♣ **Primary Outcomes** – L197
♣ **Secondary outcomes** – L205-2013

x. **Ethical Issues:** Trial registration, ethics and its intervention – L55 & L255-265

My Kind regards
